# Caregivers' assessment of meaningful and relevant clinical outcome assessments for Sanfilippo syndrome

**DOI:** 10.1186/s41687-022-00447-w

**Published:** 2022-04-25

**Authors:** Katherine Ackerman Porter, Cara O’Neill, Elise Drake, Sara M. Andrews, Kathleen Delaney, Samantha Parker, Maria L. Escolar, Stacey Montgomery, William Moon, Carolyn Worrall, Holly L. Peay

**Affiliations:** 1 Center for Genomics, Bioinformatics, and Translational Research, RTI International, Research Triangle Park, NC USA; 2 Cure Sanfilippo Foundation, Columbia, SC USA; 3 Global Patient Advocacy and Engagement, BioMarin Pharmaceutical Inc., San Rafael, CA USA; 4 Patient and Policy Affairs, Lysogene, Neuilly sur Seine, France; 5 Department of Pediatrics, University of Pittsburgh Medical Center, Pittsburgh, PA USA; 6 Children’s Hospital of Pittsburgh of UPMC, Pittsburgh, PA USA; 7 Cure Sanfilippo Foundation Parent Advocates, Columbia, SC USA

## Abstract

**Objectives:**

Sanfilippo syndrome is a rare multisystem disease with no approved treatments. This study explores caregiver perspectives on the most impactful symptoms and patient-relevant clinical outcomes assessments. The pediatric onset and progressive neurodegenerative nature of Sanfilippo limits use of self-report in clinical research. This study obtains Sanfilippo caregiver data to support the selection of fit-for-purpose and patient-relevant clinical outcome assessments (COAs).

**Methods:**

We conducted an asynchronous online focus group (n = 11) followed by individual interviews with caregivers (n = 19) of children with Sanfilippo syndrome. All participants reported on the impact of disease symptoms and level of unmet treatment need across Sanfilippo symptom domains. Focus group participants reviewed existing assessments relating to 8 symptom domains (15 total assessments) and provided feedback on meaningfulness and relevance. Focus group data were used to reduce the number of assessments included in subsequent interviews to 8 COAs across 7 symptom domains: communication, eating, sleep, mobility, pain, behavior and adapting. Interview respondents provided data on meaningfulness and relevance of assessments. Data were coded using an item-tracking matrix. Data summaries were analyzed by caregivers’ responses regarding meaningfulness; relevance to Sanfilippo syndrome; and based on caregiver indication of missing or problematic subdomains and items.

**Results:**

Participants’ children were 2–24 years in age and varied in disease progression. Caregivers reported communication and mobility as highly impactful domains with unmet treatment needs, followed closely by pain and sleep. Domains such as eating, adaptive skills, and behaviors were identified as impactful but with relatively less priority, by comparison. Participants endorsed the relevance of clinical outcome assessments associated with communication, eating, sleep, and pain, and identified them as highly favorable for use in a clinical trial. Participants specified some refinements in existing assessments to best reflect Sanfilippo symptoms and disease course.

**Discussion:**

The identification of impactful symptoms to treat and relevant and meaningful clinical outcome assessments supports patient-focused drug development. Our results inform targets for drug development and the selection of primary and secondary outcome assessments with high meaningfulness and face validity to Sanfilippo syndrome caregivers. Assessments identified as less optimal might be refined, replaced, or remain if the clinical trial necessitates.

**Supplementary Information:**

The online version contains supplementary material available at 10.1186/s41687-022-00447-w.

## Background

Mucopolysaccharidosis III (MPS III) or Sanfilippo syndrome is a severe progressive multisystem disorder of pediatric onset that has no approved therapy [1–3]. This rare and fatal condition is characterized by developmental impairments including progressive decline of cognitive and physical functioning. Symptoms typically come to attention by the time a child reaches 2–6 years of age, though symptom severity, onset and rate of disease progression is heterogeneous within and between all four sub-types (A–D) [4–7]. The most common presenting findings include impairment in speech and motor function [4, 8], with progressive cognitive decline [2]. Behavioral disturbances are frequent in young children with Sanfilippo syndrome, particularly behaviors that impact the safety of themselves and others [9, 10], and the inability to adapt to changes [1, 2, 10, 11]; these symptoms are reduced with disease progression [1, 8, 11]. Other features include acute pain, headaches, gastrointestinal pain, and pain in the joints, as well as ill-defined episodes of distress [12, 13]. Sleeping challenges, including restlessness and frequent waking at night, are also common [14]. In later stages of the disease, children’s declining oral-motor skills result in dysphagia and problems with aspiration, often requiring placement of a feeding tube [15].

Clinical outcome assessments (COAs) should be based on parameters that matter to patients [16]. In drug development, there is an increasing focus on assessing patient and caregiver experiences and preferences [17]. The Food and Drug Administration (FDA) developed guidance on collecting patient input and draft guidance on methods to identify what is important to patients, each with the objective of informing treatment development [18, 19]. The guidance addresses the significance of exploring patient perspectives on meaningful endpoints and assessments, in part to increase likelihood for enrollment and retention in clinical trials [19].

The FDA also provides guidance on selecting or modifying fit-for purpose COAs. Patient reported outcome measures (PROMs) are a type of COA that provide a standardized method for collecting data directly from patients [20]. Due to the variations in phenotype and timing of presentation in many disorders, there may be challenges with application of PROMS to specific disease populations; this challenge is particularly salient for rare disease communities [21, 22], including Sanfilippo syndrome. The inability of patients to self-report due to their young age or the cognitive impact of disease progression is coupled with the challenge of applying existing, validated assessments to the highly specific context of a multisystem and progressive disorder. Thus, caregiver input is essential when developing, validating, and selecting measures for use in clinical research [23]. Our prior research on caregivers of children with Sanfilippo syndrome resulted in a set of highly impactful symptoms that were prioritized as new treatment targets [24, 25]. The objective of this study was to extend our prior research to support the selection of fit-for-purpose and patient-relevant clinical outcome assessments for prioritized symptoms.

## Methods

### Study design

We conducted a two-stage study. The first stage, an asynchronous focus group of caregivers, was conducted to confirm the impact of the symptom domains identified in our earlier work [24] and to explore the meaningfulness and relevance of COAs to assess those symptoms. Asynchronous focus groups provide an efficient and cost-effective method that enables data collection from multiple caregivers [26–28]. The resulting data allowed the research team to reduce the number of assessments included in the second stage. The second stage comprised one-on-one interviews with a different group of caregivers of children with Sanfilippo, with similar aims of confirming the impact of the symptom domains and obtaining detailed data about the relevance and meaningfulness of the reduced set of COAs. Caregivers provided feedback on the assessments individually and were not asked to consider the potential caregiver burden of a set of assessments.

This investigation was sponsored by Cure Sanfilippo Foundation. All steps in the research process were informed by a multi-disciplinary advisory committee of researchers, physicians, and parents. Eight symptom domains related to Sanfilippo syndrome were identified from our prior data [24, 25, 29]. Across the domains, 34 COAs were initially identified by researchers, clinicians, and parents on the Advisory Committee, and then reduced to 15 by Advisory Committee through consensus based on perceived relevance to Sanfilippo clinical research (see Table 1). For each symptom domain, Advisory Committee members nominated relevant COAs in writing. Next, the list of domains and COAs were provided to the Advisory Committee to “vote” on the most relevant COAs based on relevance and fit-for-purpose items based on their clinical expertise and caregiving experiences. The Advisory Committee met with the researchers to develop the final list to ensure each symptom domain included at least one relevant COA for the study. The resulting assessments included 14 PROMs obtained via parent proxy and two clinician reported measures (for mobility). Written approvals were obtained by all licensing agents prior to using the instruments in this exploratory manner. None of the COAs were developed specifically for measuring outcomes in Sanfilippo syndrome, but all had been used in clinical trials for Sanfilippo and/or other disease indications.

**Table 1:** Prioritized symptom domains and related clinical outcome assessments

Domain	Clinical outcome assessment and description	Number of interviewees reporting on COA
Communication and relationships	*Vineland Adaptive Behavior Scales-3 Parent/Caregiver Form: Listening and Understanding* Caregiver-reported measure of attending, understanding, and responding appropriately to information from others [30]	9
	*Vineland-3 Parent/Caregiver Form: Talking* Caregiver-reported measure of using words and sentences to express oneself verbally to others [30]	9
	*Vineland-3 Parent/Caregiver Form: Relating to Others* Caregiver-reported measure of responding and relating to others, including friendships, caring, social appropriateness, and conversation [30]	Not used for interviews
Eating/swallowing	*Child Oral and Motor Proficiency Scale (ChOMPS)* Caregiver-reported assessment of eating, drinking, and related skills in children 6 months to 7 years of age [31]	10
Sleep	*Children’s Sleep Habits Questionnaire (CSHQ)* Caregiver–reported questionnaire to examine sleep behavior in young children [32]	10
	*Sleep Diary* Caregiver-reported diary completed daily to gather information about sleep patterns	Not used for interviews
Getting around	*Timed Four Stair Climb* Clinician assessed time to climb 4 standard-sized stairs [33]	19
	*Floor to Stand Test* Clinician assessed time to stand from a supine position [33]	Not used for interviews
	*Vineland-3 Parent/Caregiver Form: Using Large Muscles* Caregiver-reported physical skills in using arms and legs for movement and coordination in daily life [30]	Not used for interviews
	*Vineland-3 Parent/Caregiver Form: Using Small Muscles* Caregiver-reported physical skills in using hands and fingers to manipulate objects in daily life [30]	Not used for interviews
Pain	*Non-Communicating Child’s Pain Checklist-Revised (NCCPC-R)* Caregiver-reported pain measurement tool specifically designed for children with cognitive impairments [34]	9
Behavior	*Aberrant Behavior Checklist (ABC)* Caregiver-reported problematic behaviors for people with developmental disabilities [35]	10
	*Vineland-3 Parent/Caregiver Form: Caring for Home* Caregiver-reported assessment of household tasks such as cleaning up after oneself, chores, and food preparation [30]	Not used for interviews
Adapting	*Vineland-3 Parent/Caregiver Form: Adapting* Caregiver-reported behavioral and emotional control in new/different situations involving other people [30]	9
Caring for self	*Vineland-3 Parent/Caregiver Form: Caring for Self* Caregiver-reported self-sufficiency in such areas as eating, dressing, washing, hygiene, and health care [30]	Not used for interviews

Column 1 shows the 8 symptom domains included in this study

Column 2 represents the 15 assessments assessed by all the participants in the asynchronous focus group and provides a description of the outcome assessment

Column 3 shows the number of interviewees who reviewed each COA that was selected for the interviews

### Inclusion criteria and participant recruitment

Participation was available to English-speaking parents or guardians with primary caregiving responsibilities of a living child diagnosed with Sanfilippo syndrome. Caregivers were eligible to participate in only one of the two study activities. For the asynchronous focus group, participants were required to have access to a Facebook account (i.e., either use an existing account or create a new account). For one-on-one interviews, internet video conferencing was the preferred platform, though a phone interview was an offered alternative.

Cure Sanfilippo Foundation recruited participants through the ConnectMPS Registry and via snowball sampling through existing formal and informal parent support networks. Online consent was obtained from all participants. Both activities were determined to be exempt from review by the RTI International Institutional Review Board.

### Data collection methods: activity 1—asynchronous focus group

Participants first completed an online survey on demographics, parent-reported physician diagnosed sub-type of Sanfilippo syndrome, and symptoms. Asynchronous focus group data collection occurred over a consecutive 5-day period in October 2019 using a private Facebook group. The acceptability of using social media platforms for data collection is included in the Patient Focused Drug Development guidance [18, 19]. Online engagement with and between participants was moderated by KAP using a semi-structured focus group guide (available as Additional file 1). Activities consisted of: (1) responding to written queries regarding symptom impact, and (2) reviewing and commenting on COAs relating to each domain. Participants responded to polls embedded in the moderator’s Facebook posts regarding the level of difficulty a child experiences with the symptoms and the level of importance to treat the symptom. All questions were based on a 5-point Likert scale. The first 4 days of this activity included prompts designed to assess attitudes from all participants regarding the eight symptom domains and 15 associated COAs. All participants were prompted to expand on previous comments by the investigator during data collection. The final day provided participants additional time to respond to prompts added by the investigator for clarification and depth (see Additional file 1), and to comment on others’ posts from previous days. Participants had varying levels of engagement throughout the focus group; some provided more prolific responses than others and some engaged more with the embedded polls than with open-ended question prompts. Table 1 displays the COAs used for this activity.

### Data collection methods: activity 2—one-on-one interviews

Interview participants first completed the same online survey that was completed by focus group participants. Interviews were then conducted via videoconference and lasted between 60 and 90 min. Interviews were completed from November to December 2019. Practices identified by International Society of Pharmacoeconomics and Outcomes Research (ISPOR) were used to guide data collection on meaningfulness and relevance of the outcome assessments to Sanfilippo syndrome, including exploration of the face validity of each COA [18, 19].

See Table 1 for the COAs included in this activity. To keep the interviews to a feasible length, each participating caregiver was assigned to respond to four symptom domains. Domains were selected to be relevant to each participant’s child based on their responses to the symptom survey. Either nine or ten participants reviewed each measure, except for the four-stair climb, which all 19 participants reviewed since it was the only clinical assessment shown in the interview phase and was very brief. Symptoms and domains were presented to participants in a standardized order. Prior to the interviews, participants received the instruments via email to allow for an optional review period.

A semi-structured interview guide (available as Additional file 2) was developed to conduct the interviews and tailored to each participant. For each of the four symptom domains addressed in each interview, participants first responded to questions about the impact of associated symptoms on their child and perceived unmet treatment needs related to those symptoms (e.g., for communication, caregivers were asked first about the impact of impairments in reciprocal communication on their child and to identify specific examples). They then reported how important it would be for their child to participate in a clinical trial that assessed the domain. Next, participants reviewed the COA associated with each domain and responded about each assessment’s meaningfulness and relevance to Sanfilippo syndrome symptoms, and any missing or problematic elements or items. Finally, they reported on their attitudes about the ability of the COA to show a meaningful change during a clinical trial.

### Data analysis

#### Asynchronous focus group

Survey data collected on the symptoms were analyzed descriptively. The child’s Sanfilippo stage was estimated as early/mid stage or later stage based on a combination of caregiver-reported cognitive ability, self-feeding ability, and autistic behaviors. Children rated by caregivers as having poor cognitive ability and who were also either (1) fed by someone else or tube-fed or (2) exhibiting autistic behavior “always” or “almost always” were identified as later stage.

Focus group data were manually entered into a summary item-tracking matrix [36] that was organized by domain and COA to allow for a rapid analytic approach [37, 38]. One investigator summarized responses for every participant and across each domain in the matrix. Findings were synthesized into a report that was reviewed and validated by cross-referencing data with the matrix by the senior investigator. Given the concept reduction aim of the focus group, analysis focused on: (1) verifying that the symptom domains were impactful and associated with unmet treatment need, (2) the meaningfulness and relevance of the assessments for use in a clinical trial setting, and (3) the feedback on items or procedures in the assessments.

An objective of the focus group was to reduce the number of COAs for the interview phase. The selection of COAs for use in interviews occurred via a consensus between RTI investigators and the advisory committee, which included members of the Cure Sanfilippo Foundation. COAs were narrowed to those that represented more patient-focused and disease-relevant tools.

#### One-on-one interviews

Structured interview notes were developed from recorded one-on-one interviews and compiled in a summary item-tracking matrix. The matrix was organized by symptom domain, COA, and items within each assessment [39]. The matrix was coded separately by two investigators who conducted the interviews using a rapid analysis approach [36, 40]. Each interviewer summarized the data in the matrix by domain impact and unmet need; by COA measure, subdomain, and item meaningfulness and relevance to Sanfilippo syndrome. Discrepancies were resolved through a consensus between the interviewers and the senior investigator. Findings were synthesized into a report that was reviewed, along with the matrix and data summaries, by the senior investigator.

Survey data collected on the symptoms were analyzed descriptively. The child’s Sanfilippo stage was estimated using the same approach described above. Table 1 displays the 8 domains covered in this study. The final column includes the number of participants reporting on the measure during the interviews.

## Results

### Activity 1: asynchronous focus group

For the asynchronous focus group activity, all participants were biological mothers (n = 11). One participant did not complete the pre-interview survey, and self-reported as a biological mother during the group. The median age was 35 years. All participants self-identified as White, non-Hispanic/Latina, as indicated in Table 2. Participants’ children with Sanfilippo syndrome had a median age of 7.5 years. Most children were diagnosed with Sanfilippo syndrome sub-type A (n = 9), and most were defined as Early/Mid Stage (n = 7). See Table 3.

**Table 2:** Asynchronous focus group participant demographics from survey (n = 10)

	Median	Range
Age (years)	35	(32–45)
	Count	
Countries		
USA	8	5 states
Australia	2	2 states/territories
	Count	% of respondents
Marital Status		
Married/Partnered	8	80%
Single	1	10%
Separated/divorced	1	10%
Sex		
Female	10	100%
Relationship to child		
Biological mother	10	100%
Race		
White	10	100%
Education		
Did not finish HS	1	10%
High school graduate	1	10%
Some college	2	20%
Bachelor’s degree	3	30%
More than a bachelor’s degree	3	30%
Income		
Less than $25,000	2	20%
$100,000–$149,000	5	50%
$150,000 or more	2	20%
Unanswered	1	10%

n = 11 (data missing from 1 participant)

**Table 3:** Characteristics of child with Sanfilippo syndrome: focus group survey (n = 10)

	Median	Range
Age (years)	7.5	3–10
	Count	% of respondents
Sex		
Male	5	50%
Female	5	50%
Sub-type		
A	9	90%
B	1	10%
Staging		
Early/mid stage	7	70%
Later stage	3	30%
Participated in a clinical trial	5	50%

n = 11 (data missing from 1 participant)

Most participants reported high impact of all eight symptom domains (Table 1) and associated high unmet treatment need. Eight of the 15 COAs were preferred by caregivers based on relevance of the overall assessment and the specific items to Sanfilippo syndrome. For domains with more than one measure, caregivers compared the items and identified the assessment that was most relevant to the characteristics and lived experience of Sanfilippo syndrome. Though there was not complete agreement on all assessments, participant report on meaningfulness and relevance of the COAs to Sanfilippo syndrome permitted the identification of more and less prioritized assessments. In addition, when responding to prompts used in the focus groups, participants were able to inform ways the existing COAs could more accurately capture their child’s symptoms, for example addressing the time window for reporting (e.g., “in the last 7 days…”) or adding items specific to Sanfilippo-related symptoms (e.g.., overfilling of mouth when eating). In addition, respondents identified COAs that had favorable justifications for use at specific stages of Sanfilippo progression (e.g., assessments deemed meaningful when the child had better function, but not now). The outcome assessments identified as less preferred by participants were omitted from Activity 2.

### Activity 2: one-on-one interviews

All participants who completed one-on-one interviews (n = 19) were biological parents with a median age of 38 years. Most participants self-identified as White (n = 17). Participants’ children ranged in ages of 2–24 years, with a median age of 9 (Table 4). They included Sanfilippo subtype A (n = 13), subtype B (n = 5), and subtype C (n = 1). Eleven children (56%) were in the later stage of Sanfilippo syndrome, and 8 children (42%) were in the early/mid stage, as depicted in Table 5.

**Table 4:** Interview participant demographics

	Median	Range
Age (years)	38	(29–54)
	Count	
Countries		
USA	19	13 states
	Count	%
Marital Status		
Married/partnered	17	89%
Single	2	11%
Sex		
Male	2	11%
Female	17	89%
Relationship to child		
Biological father	2	11%
Biological mother	17	89%
Race		
African America/Black	1	5%
Asian	1	5%
White	17	89%
Education		
Did not finish HS	0	0%
High school graduate	1	5%
Associates degree	2	11%
Some college	3	16%
Bachelor’s degree	6	32%
More than a bachelor’s degree	7	37%
Income		
Less than $25,000	2	11%
$25,000–$49,000	2	11%
$50,000–$74,999	2	11%
$75,000–$99,999	2	11%
$100,000–$149,000	3	16%
$150,000 or more	8	42%

n = 19

**Table 5:** Characteristics of oldest child with Sanfilippo syndrome: interviews

	Median	Range
Age (years)	9	(2–24)
	Count	%
Sex		
Male	10	53%
Female	9	47%
Sub-type		
A	13	68%
B	5	26%
C	1	5%
Staging		
Early/mid stage	8	42%
Later stage	11	58%
Participated in a clinical trial	6	32%

n = 19

### Symptom domains

#### Communication

Nine participants reported on the communication domain as it relates to their child with Sanfilippo syndrome. All participants indicated challenges with communication, with most describing verbal communication capabilities as highly important. Eight stated their child’s communication was declining. Five caregivers described ways in which they communicate with their non-verbal children, such as by using speech generating devices or sign language. Overall, this domain was considered highly impactful and represents a significant unmet treatment need. Only one parent indicated reduced importance of treating communication challenges based on the child’s severe stage of disease progression. All caregivers placed a high value on even a small improvement in any form of communication ability that could come from a new treatment.

#### Eating/swallowing

Ten participants reported on the eating/swallowing domain. All caregivers reported their children were able to eat without a feeding tube, but six required at least some assistance. Five caregivers reported their children faced challenges with choking from eating too quickly or overfilling their mouths and expressed a need to monitor their child while eating. Overall, this domain was described as a high impact symptom by many caregivers. Four participants indicated other domains were currently more important (specifically, sleep and communication) but they anticipated additional unmet needs related to eating and swallowing as their child’s symptoms progress. For most caregivers, value was placed on even a small improvement to eating and swallowing that could come from a new treatment. One parent reported that a larger change, resulting in independent eating would be needed to significantly improve the child’s quality of life.

#### Sleep

Ten participants reported on the sleep domain. Major issues reported for these children with Sanfilippo syndrome included: having difficulty falling asleep, waking up and getting out of bed in the middle of the night, not sleeping for several nights in a row, and having night-time seizures. Sleep was reported as not only a challenge for the child with Sanfilippo syndrome, but also for family members whose sleep was disrupted by the child. Eight participants endorsed sleep as a very important issue at some point in their child’s history; some indicated that it is less of an issue right now, either due to sleep medication regimen or disease progression, and therefore not a current treatment priority for their child. Four participants indicated that a small improvement, such as an extra hour of sleep or reducing the need for sleep medication, would make a meaningful impact at the time(s) in their lives when their child’s sleep was disrupted.

#### Mobility

All 19 participants reported on the mobility domain. Participants reported a range of mobility levels in their children, from highly mobile (jumping, running) to full-time wheelchair use. Seven children require mobility assistance for safety or due to regression associated with mobility, such as fatiguing more quickly or needing to take stairs one at a time. Fourteen participants reported this domain as very important due to impact on independence and caregiving burden. Nine participants indicated that only a small improvement in mobility, such as the ability to transfer between positions or the capacity to use stairs, would make a meaningful impact.

#### Pain

Nine participants reported on the pain domain. Four participants reported their child having a high pain tolerance, while some acknowledged their uncertainty about whether their child experienced pain. Six participants indicated that the child’s communication impairments made it difficult to know when a child is in pain versus some other cause of distress. Four participants reported facial expressions or sounds expressed by the child as a primary indicator of pain. Pain was reported to be a highly important domain, though somewhat challenging for participants to interpret. Three caregivers reported that a small improvement in pain would make a meaningful impact, while six indicated that the ability to better communicate feeling pain would be more meaningful.

#### Behavior

Ten participants reported on the behavior domain. Three reported behaviors as a problem when their children were younger but not any longer. For the seven participants reporting behavior challenges, they described a variety of behaviors, such as aggression, over-excitement, inappropriate responses in social settings (such as screaming or lunging), and safety risks associated with impulsivity. Six participants indicated that behavioral issues tend to be a problem in public and for safety. One participant indicated this is the area that affects the family the most and causes caregivers to lose patience. Three participants reported that their child's behavior was not a problem that needed to be treated. For example, one participant stated: “That’s how my child expresses [him/herself].” For the participants who viewed their child’s behavior as problematic, even small improvements would be meaningful as a treatment outcome.

#### Adapting

Nine participants reported on the adapting domain. When asked about adaptation to new routines and environments, six caregivers reported their children had minimal issues with adapting as their child got older. Five participants indicated that adapting to new environments, such as changes in transitioning routines, was less important to treat compared to other domains, particularly as their children have experienced more severe progression.

### Clinical outcome assessments

#### Communication, as measured by the Vineland-3 parent/caregiver form: listening and understanding; and the Vineland-3 parent/caregiver form: talking

Participants reported positive perceptions of the meaningfulness and relevance of both Vineland domains, *Listening and Understanding* and *Talking* [30]. They endorsed a clinical trial focused on improving communication that uses this COA, though they found the *Listening and Understanding* domain to be slightly more meaningful and acceptable than the *Talking* domain. Most participants reported that these Vineland assessments captured meaningful and relevant verbal and non-verbal items across different stages of Sanfilippo syndrome, though participants of younger children and those with more verbal ability placed higher value on this assessment than caregivers of children with less verbal ability. Two participants reported that because their children’s communication abilities were poor, their priority was for the children to convey basic needs in a simple way, and therefore some of the items were less relevant. They also stated that the items did not permit assessment of their child's peak ability level (earlier in disease progression). Five suggested measuring non-verbal items, such as facial expressions or gestures, to communicate basic needs. For *Vineland-3 Parent/Caregiver Form: Listening and Understanding*, some participants reported challenges in responding to the items because they could not rate their children’s receptive language.

#### Eating and swallowing, as measured by the Child’s Oral and Motor Proficiency Scale

Many participants reported overall positive perceptions of the *Child’s Oral and Motor Proficiency Scale* (ChOMPS) [31] as a meaningful COA. Four indicated the exception is when the child progresses to the exclusive use of a feeding tube, at which time participants expressed poor relevance to their child’s situation and insufficient ability of the assessment to document change in non-oral feeders. Several participants also advocated for adding an item regarding the child overfilling his/her mouth as an important symptom-specific feature of Sanfilippo syndrome. Additionally, participants provided feedback on response items, suggesting that a scale that included current status as well as whether the item was ever attained would better capture peak abilities and subsequent regression.

#### Sleep, as measured by the Children’s Sleep Habits Questionnaire

Participants also reported positive perceptions on the *Children’s Sleep Habits Questionnaire* (CSHQ) [32]. Though almost all participants stated this was a meaningful and relevant assessment for clinical trials focused on improving sleep, many indicated some critical items were missing or had insufficient specificity to capture the Sanfilippo syndrome experience. An example is the impact of sleep medication on how participants would respond to the measure. Several participants indicated some items may be more challenging for participants whose children are non-verbal, particularly questions about feeling fearful of the dark or awakening due to a bad dream.

#### Mobility, as measured by the Timed Four Stair Climb

Participants revealed mixed perceptions about the *Timed Four Stair Climb* [33]. Most participants reported that this measure should be adapted or include additional mobility assessments. Although they identified the ability to climb stairs was a valuable activity, many participants reported that the speed to climb stairs was not the most important indicator of motor skills in their children. Other recommendations for meaningful measurement included measuring stability on stairs, use of alternating feet on stairs, assessment of quality of running/walking, ability to independently rise from a sitting position, and ability to maneuver around objects. Additional concerns were expressed by all participants regarding cognitive and behavioral factors that would impact the child’s ability to follow directions to successfully complete the stair climb. A global, multi-domain measurement of mobility would be preferred by most participants.

#### Pain, as measured by the Non-communicating Child’s Pain Checklist-Revised

Participants reported the *Non-Communicating Child’s Pain Checklist-Revised (NCCPC-R)* [34] as moderately meaningful to measure in a clinical trial but had concerns about the assessment’s relevance and specificity to Sanfilippo symptoms. There was almost universal concern about the reporting timeframe of 2 h (as indicated in the measure instructions). Many participants recommended a timeframe of at least 1 week to capture the child’s more chronic and diffuse pain symptoms. Further, some participants reported that their child displays features assessed in the measure, but they were unsure if their children’s reactions were in response to pain or to some other stimuli. Some participants advised including other components, such as the site of the pain: e.g., gastrointestinal pain, headaches, and joint pain. Two others reported the importance of understanding the underlying cause of pain, which is not assessed in the measure. Participants concluded that with a few adaptions, this measure would be highly meaningful and relevant as a clinical trial outcome for Sanfilippo syndrome.

#### Behavior, as measured by the Aberrant Behavior Checklist

Most participants reported positive perceptions of many of the items in the *Aberrant Behavior Checklist (ABC)* [35] but expressed some concerns about relevance and specificity to Sanfilippo syndrome. Some items were described as unclear in the Sanfilippo context; for example, an item asks if their child "refuses to follow directions.” Many children were described as unable to follow directions but not actively disobeying their participants, and thus participants expressed uncertainty about responding to this question. Some participants reported that there were important missing items including positive behaviors such as laughing, or other topics such as putting non-food items in mouth and inappropriate food-seeking behavior, fleeing and other safety issues, and grinding teeth. Some participants identified some items as repetitive. Several participants reported being displeased by the negative and problem-focused nature of the scale.

#### Adapting, as measured by the Vineland 3 Parent-Caregiver Form: Adapting

Participants reported mixed perceptions on *Vineland-3 Parent/Caregiver Form: Adapting* [30]. Some participants reported this measure could be meaningful in a clinical trial aimed at improving adaptation in children with Sanfilippo syndrome, especially in young children, but some items were reported as not applicable, especially as symptoms progressed. For example, four participants reported that some of the items about verbal communication would not apply to their children. Additionally, some participants noted items that would require a higher level of cognitive function than their children experience (e.g., “refuses to follow directions” would suggest that they child is disobeying the parent, while the child may not be able to process directions given). One participant perceived that the items in the measure did not represent a cohesive domain. Some suggested that additional items were necessary on child reactions to changes in routine.

**Fig. 1:**
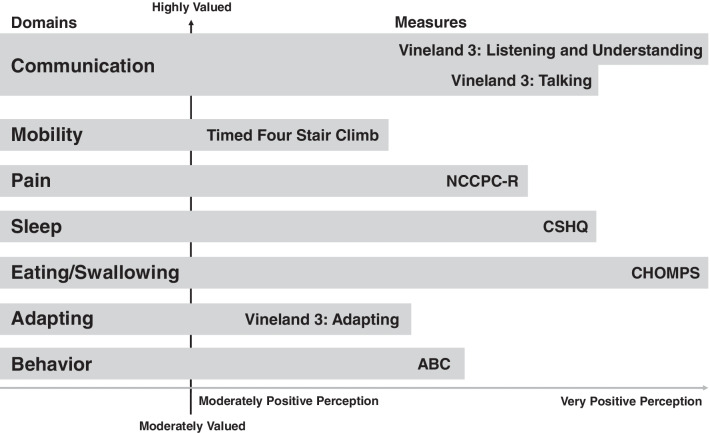
Comparison of caregiver valuing of outcome measure symptom domain and associated

A comparison of symptom impact and importance and the instrument used to measure the outcome domain can be found in Fig. 1.

## Discussion

This study of caregivers provides data on impactful symptoms of Sanfilippo syndrome and the selection of fit-for-purpose and patient-relevant COAs for those symptoms. We employed a pragmatic approach to obtain data in a rare disease population. The asynchronous focus group was a feasible method for obtaining preliminary data to confirm the symptom domains and reduce the number of COAs used in the interviews. This allowed us to have a reasonable sample size for the interviews and a feasible interview length, without as much concern about leaving out important COAs. Though the severity of the disease and associated cognitive impairments limited our study to proxy caregiver reports, this approach could also be used for self-report on impact and relevance in other disease indications.

Overall, our data support the importance of diverse symptom domains that extend beyond cognitive. Participants reported that all of the symptom domains included in the study represented meaningful areas of high impact and unmet treatment need; this outcome was consistent with our prior research with Sanfilippo caregivers [25, 29]. The most highly impactful domains were communication and mobility, followed by pain and sleep, while domains of adapting, behavior, and eating/swallowing were noted as meaningful but with relatively lower impact by our parent participants. The progressive nature of Sanfilippo syndrome resulted in some symptoms being more impactful at different times in disease progression.

Among the outcome assessments, participants highly regarded the *Vineland-3 domain Parent/Caregiver Form: Listening and Understanding, ChOMPS*, the *CSHQ,* and the *NCCPC-R* due to meaningfulness and perceived ‘fit’ with their experience as caregivers. Participants indicated high relevance and face validity at various stages of disease progression. Participants reported that the child’s and family’s quality of life was fundamental in determining meaningfulness and relevance of the assessments. Additionally, participants indicated items within COAs that were less relevant and less meaningful based on Sanfilippo symptoms and disease course. Participants commonly remarked upon the challenge of accounting for the regression in milestones that is a hallmark of Sanfilippo syndrome, and they desired question sets and response options that permit the capture of declining function.

Figure 1 depicts the symptom domain and COA prioritization. There are several instances where a symptom domain was highly valued, but an associated COA was not equally endorsed. An example is mobility, a highly impactful symptom domain where the measure selected for this study was not determined to be highly relevant to this condition. There are many other mobility assessments that may be relevant at specific stages of Sanfilippo syndrome progression that could be explored and validated in this population.

This study provides evidence for impactful symptoms with unmet treatment need, and for the meaningfulness and relevance of associated COAs for use in clinical trials for Sanfilippo syndrome. While the selection of assessments utilized in a clinical trial depends on a range of factors that extend beyond caregiver preferences, those developing trial protocols should consider the integration of these highly relevant and meaningful COAs as primary or secondary outcome measures. For many of these outcomes, additional natural history for children with Sanfilippo syndrome would be useful to inform the customization and validation of Sanfilippo-specific forms. Our data support what is important to caregivers and reflective of their lived experience, but further exploration of meaningful change in the context of a clinical development program is needed. Further, our study supports the practice of transparently explaining the purpose behind selecting outcome measures, which can be helpful in fostering an engaged trial cohort throughout the entirety of the trial follow up.

### Limitations

Data collection was limited to English-speakers and all interviewed participants were from the United States. Perspectives from other countries may provide important additional attitudes. Our sample did not include all the Sanfilippo syndrome sub-types. While we sought breadth and heterogeneity in respondents, our study cannot account for the many factors underlying caregiver experiences and preferences. Based on the study approach, we could make only a basic assessment of saturation in responses. Future research should include larger numbers of caregivers representing sub-types C and D to further explore nuances between all four sub-types, though this presents a significant challenge in ultra-rare disease populations. Additionally, this study recruited using a convenience sample accessed by a disease-focused foundation. Future studies would be strengthened by the inclusion of a representative sample.

Using an asynchronous focus group via Facebook allowed us to systematically collect data from caregivers remotely, conveniently, and in a timely manner; however online participants tended to provide brief comments using fewer words of agreement or disagreement compared to the subsequent in-person interviews. These limitations were reduced by pairing the focus group with interviews and using the focus group data primarily to reduce the set of outcome assessments to a feasible number for interviewees. The order of the data collection did not vary between respondents, and in this exploratory qualitative study we cannot determine if there was an order effect based on the sequencing of the assessments.

Some participants with further progressed children were asked to “think back” to earlier in the disease progression when considering symptom impact. This may result in recall bias. Subsequent studies that utilize clinical staging in a larger sample would allow a statistical assessment of differences by stage.

Finally, the research team selected COAs (and in the case of the Vineland-3, selected domains of a global measure) to match symptom domains that were indicated as particularly impactful in prior studies. For many of the symptom domains, there were multiple COAs that we could have selected, and each outcome assessment has strengths and weaknesses. Future research should assess a broader set of assessments as potentially relevant and impactful in the Sanfilippo context while also taking into consideration fit-for-purpose and responsiveness to change in a clinical trial setting.

These data should be interpreted as informing unmet symptom needs and COAs that may be considered as more or less meaningful to caregivers in a clinical trial context instead of an endorsement of a particular outcome measure. Additional research could also explore potential burden of using a battery of outcome assessments such as these for clinical trial use; however, this was outside the scope of our study.

## Conclusion

Like many rare diseases, there are few existing COAs specific to Sanfilippo syndrome. Ideal COAs should be meaningful to caregivers (and thus anticipated to be meaningful to patients), relevant and fit-for-purpose, and applicable in the context of a clinical development program. Current trials include COAs that measure many of the symptom constructs that were identified as impactful by the caregiver participants in this study. Existing clinical development programs may consider evidence generation and validation efforts to support these data. New clinical development programs could consider these results to select, modify, and further validate COAs. Trial developers may also use this data to select a multi-domain or composite approach to measuring outcomes in Sanfilippo syndrome.

## Availability of supporting data

This study collected in-depth qualitative data from caregivers of children with a very rare disease, and thus our data is extremely difficult to de-identify. The informed consent signed by our participants specifies that their data will be kept confidential and that only aggregated data and illustrative quotes will be reported. Thus, the raw data from this study will not be accessible.

## Supplementary Information

**Additional file 1.** The online asynchronous focus group guide was used for data collection for study activity 1 and included prompts over 5 days.

**Additional file 2.** The interview guide was used for data collection for study activity 2 and included the exploration of 4 pre-selected domains for each participant.

## Abbreviations

MPS III: Mucopolysaccharidosis III
COA: Clinical Outcome Assessment
FDA: Food and Drug Administration
PROM: Patient Reported Outcome Measure
PRO: Patient Reported Outcome
ISPOR: By International Society of Pharmacoeconomics and Outcomes Research
ChOMPS: Child’s Oral and Motor Proficiency Scale
CSHQ: Children’s Sleep Habits Questionnaire
NCCPC-R: Non-Communicating Child’s Pain Checklist-Revised
ABC: Aberrant Behavior Checklist
IRB: Institutional Review Board
