# Supplementary material for: Caregivers' assessment of meaningful and relevant clinical outcome assessments for Sanfilippo syndrome
Source: J Patient Rep Outcomes. 2022 Apr 25;6:40. doi: 10.1186/s41687-022-00447-w (PMC9038975; doi:10.1186/s41687-022-00447-w)
Supplement: Supplementary file 1 — Additional file 1. The online asynchronous focus group guide was used for data collection for study activity 1 and included prompts over 5 days. [file 41687_2022_447_MOESM1_ESM.docx]

**S1. Facilitator’s Online Focus Group Guide**

**Duration: up to 1/2 hour per day for six days (~2.5 hours total)**

# Asynchronous Focus Group Schedule

| **Monday, 10/21** | **Tuesday, 10/22** | **Wednesday, 10/23** | **Thursday, 10/24** |
| --- | --- | --- | --- |
| Communication | Eating/Swallowing  Sleep | Mobility  Pain | Behavior |

# Daily Post

- **Morning post**: *Today we’re going to talk about [domain].**?* [Prompt parent to respond Using a Facebook poll and a 5-point Likert-type scale on the impact of domain on child] *On a scale of ‘very easy’ to ‘very difficult’*

*Share some examples of how challenges with [domain]with your child affects your day*. [open]

*How important is a new treatment that could improve [domain]?*

*[5-point Likert-type scale]*

*Would you need to see a big difference in [domain] for the new treatment to be ‘worth it,’ or would a small difference be enough? [open]*

- **Afternoon post**: Provide outcome measure and probing questions for participants’ responses.

*Imagine a clinical trial for Sanfilippo syndrome. The goal of the trial medication is to improve[domain]. Before, during, and after the trial researchers will assess [domain] in your child.*

*There are different ways that researchers can measure [domain]n, and here is one example [share outcome measure with group – either by link or upload pdf]. This would be [answered by the parent about their child/answered by a researcher].*

*Please do not answer this survey. We want you to look at the questions and response options and think of answering it for your child.*

*Read the measure and share your thoughts.*

1. *Does this measure, on the whole, represent something meaningful and important to you?*
2. *Are there specific items that you find misleading, difficult to understand, or that don’t seem to fit for a child with Sanfilippo syndrome?*
3. *Is there anything missing that would be essential to include in this question set?*

*[As relevant, add one or two additional measures with the same question set]*

- **Evening Summary Post:** Summarize the day’s findings

*Thank you for participating today.*

[Summarize day’s findings]

*Please add anything important about these measures that I may have missed.*

*Even though we are starting on a new topic tomorrow, you may continue to make comments on these posts throughout the week if you think of anything else to add.*
