# Supplementary material for: Caregivers' assessment of meaningful and relevant clinical outcome assessments for Sanfilippo syndrome
Source: J Patient Rep Outcomes. 2022 Apr 25;6:40. doi: 10.1186/s41687-022-00447-w (PMC9038975; doi:10.1186/s41687-022-00447-w)
Supplement: Supplementary file 2 — Additional file 2. The interview guide was used for data collection for study activity 2 and included the exploration of 4 pre-selected domains for each participant. [file 41687_2022_447_MOESM2_ESM.docx]

# S2 Appendix: Interview Guide: Sanfilippo Caregiver Study

Tell me a bit about your child with Sanfilippo syndrome.

Tell me about the best experience you had with your child with Sanfilippo in the past week.

# Reviewing Measures Activity

Let’s start by imagining a clinical trial. The goal of the trial is to improve a specific symptom of Sanfilippo—so it’s not a cure. We want to learn from you how important different symptoms are. Then we will show you different outcome measures that could be used in clinical trials to tell whether the new medicine is working.

We want to learn your perspective of four major symptoms of Sanfilippo syndrome. We will go through them one by one.

**For each assigned domain, participants were provided the following set of questions.**

**Possible domains included:**

- **Communication**
- **Eating/Swallowing**
- **Sleep**
- **Mobility**
- **Pain**
- **Behavior**
- **Adapting**

We will first talk about[domain]. [prompt parent on how this feature impacts their child]

Share some examples of how challenges with [domain] with your child affects your day.

How important is a new treatment that could improve [domain]? Why?

Would you need to see a big difference in [domain]for the new treatment to be ‘worth it,’ or would a small difference be enough?

Imagine a clinical trial for Sanfilippo syndrome. The goal of the trial medication is to improve[domain]. Before, during, and after the trial researchers will assess [domain].

There are different ways that researchers can measure[domain], and here is one example [share outcome measure with group – either by link or upload pdf]. This would be [answered by the parent about their child/answered by a researcher].

We want you to look at the questions and response options and answer it for your child and give us feedback on the items.

1. [Orient parent to whether this is a parent-reported or clinician-reported/evaluated measure]
2. Read the measure out loud and share your thoughts as you read.
3. Does this measure, on the whole, represent something meaningful and important to you?
4. [Review item by item. Ask if there are specific items that are misleading, difficult to understand, or that don’t seem to fit for a child with Sanfilippo syndrome]
5. Is there anything missing that would be essential to include in this question set?
6. Are there questions that would not be helpful at all?
7. What do you think about the amount of time it took you to complete the questionnaire or tool?
8. Imagine again the clinical trial story. How do you feel about this measure being used to tell whether a new medicine is making a meaningful change for your child?

Thank you for answering these questions about the symptoms and measures. Now I would like to ask you some questions about the way trials can be set up.
